# Unhealthy lifestyle associated with increased risk of macro- and micro-vascular comorbidities in patients with long-duration type 2 diabetes: results from the Taiwan Diabetes Registry

**DOI:** 10.1186/s13098-023-01018-9

**Published:** 2023-03-08

**Authors:** Li-Ju Ho, Wayne Huey-Herng Sheu, Su-Huey Lo, Yen-Po Yeh, Chii-Min Hwu, Chien-Ning Huang, Chang-Hsun Hsieh, Feng-Chih Kuo

**Affiliations:** 1 Division of Endocrinology and Metabolism, Department of Internal Medicine, Tri-Service General Hospital, National Defense Medical Center, No. 325, Section 2, Cheng-Kung Road, Neihu District, Taipei City, 11490 Taiwan, Republic of China; 2 Section of Endocrinology and Metabolism, Department of Medicine, Taipei Veterans General Hospital, Taipei, Taiwan; 3 Institute of Medical Technology, College of Life Science, National Chung-Hsing University, Taichung, Taiwan; 4 Division of Endocrinology and Metabolism, Department of Internal Medicine, Taichung Veterans General Hospital, Taichung, Taiwan; 5 Faculty of Medicine, National Yang Ming Chiao Tung University School of Medicine, Taipei, Taiwan; 6 Tao-Yuan General Hospital, Ministry of Health and Welfare, Taoyuan City, Taiwan; 7 Changhua County Public Health Bureau, Changhua, Taiwan; 8 Institute of Medicine, Chung Shang Medical University Hospital, Taichung, Taiwan

**Keywords:** Unhealthy lifestyle, Vascular complications of diabetes, Taiwan Diabetes Registry (TDR)

## Abstract

**Background:**

Unhealthy lifestyle has been associated with obesity and type 2 diabetes. Whereas its association with vascular complications in patients with long-duration of type 2 diabetes is still uncertain.

**Methods:**

A total of 1188 patients with long-duration of type 2 diabetes from the Taiwan Diabetes Registry (TDR) data were analyzed. We stratified the severity of unhealthy lifestyle via scoring three factors (sleep duration <7 or >9 h, sit duration ≥ 8h, and meal numbers ≥ with night snack) and analyzed their associations with the development of vascular complications using logistic regression analysis. Besides, we also included 3285 patients with newly diagnosed type 2 diabetes as the comparison.

**Results:**

Increased numbers of factors that stand for unhealthy lifestyle were significantly associated with the development of cardiovascular disease, peripheral arterial occlusion disease (PAOD) and nephropathy in patients with long-duration of type 2 diabetes. After adjusting multiple covariables, having ≥ 2 factors of unhealthy lifestyle remained significant associations with cardiovascular disease and PAOD, with an odds ratio (OR) of 2.09 (95% confidence interval [CI] 1.18–3.69) and 2.68 (95% CI 1.21–5.90), respectively. Among individual factor for unhealthy lifestyle behaviors, we revealed that eating ≥ 4 meals per day with night snack increased the risk of cardiovascular disease and nephropathy after multivariable adjustment (OR of 2.60, 95% CI 1.28–5.30; OR of 2.54, 95% CI 1.52–4.26, respectively). Whereas sit duration for ≥ 8 h per day increased the risk of PAOD (OR of 4.32, 95% CI 2.38–7.84).

**Conclusion:**

Unhealthy lifestyle is associated with increased prevalence of macro- and micro-vascular comorbidities in Taiwanese patients with long-duration type 2 diabetes.

**Supplementary Information:**

The online version contains supplementary material available at 10.1186/s13098-023-01018-9.

## Introduction

Type 2 diabetes mellitus (T2DM) has been well recognized as an important cardiovascular risk highly associated with several vascular comorbidities. Atherosclerotic macrovascular complications affect arteries that supply the heart, brain, and lower extremities resulting in cardiovascular disease, cerebrovascular disease, and peripheral arterial occlusive disease (PAOD), respectively [1, 2]. Diabetes also frequently accompanied by several microvascular complications, namely neuropathy, nephropathy, and retinopathy [2]. In fact, vascular diseases are the leading causes of morbidity and mortality in patients with diabetes [3]. Overall, their life expectancy is about 7–10 years shorter than people without diabetes [4]. Besides, diabetic nephropathy and retinopathy are the main contributors to end-stage renal disease and blindness which further impair their quality of life and self-care ability. Despite significant progression in the newly developed anti-hyperglycemic agents, there are still considerable T2DM patients could not achieve adequately glycemic control [5]. One important and usually ignored issue is the dysregulated lifestyle behaviors that disturbed normal physical activities are commonly presented in patients with diabetes. Indeed, strategies to facilitate behavior change have been recommended in the standards of diabetes care [6] and recent American Diabetes Association (ADA)/European Association for the Study of Diabetes (EASD) consensus conducted in June 2022 also emphasized the importance of 24-h physical behaviors for management of hyperglycemia in T2DM [7]. Considering the dysregulated life behaviors might be adjustable at the early stage of diabetes, we aim to investigate their long-lasting impacts on the diabetes associated macro- and micro-vascular comorbidities, which are currently still lack of evidence.

Unhealthy lifestyle mediated disruption of circadian rhythm is known to have lots of deleterious impacts on health [8]. For example, using the recommended 7–9 h sleep duration in adults [9] as the reference group, sleep deprivation was associated with increased risk for general obesity and abdominal obesity [10]. Likewise, ≥ 8 h of sitting time per day has been shown positively associated with incident diabetes in obese women [11]. Regarding to the eating pattern, a delayed eating schedule [12] or nighttime snacking [13] can trigger detrimental influence on the body metabolism. Disturbed circadian rhythm might influence the vascular system as well. Using cardiac magnetic resonance to define the post-reperfusion infarct size in patients with ST-segment elevation myocardial infarction, Zhao et al. [14] recently disclosed individuals with shift work had augmented reperfusion injury. Similar finding was observed in the ischemic stroke animal model, female rats underwent early exposure to shifted light–dark cycles presented elevated circulating cytokines with greater post-stroke mortality [15]. Based on these reports, unhealthy lifestyle that disturb day-night cycles very likely impedes the normal vascular regulation as encountering atherosclerotic vascular disease [16]. However, direct evidence linking unhealthy lifestyle behaviors to the diabetes associated vascular diseases is still very limited. Therefore, in this project, we utilized the data obtained from the Taiwan Diabetes Registry containing detailed assessment of the medical documentation to explore this issue.

The Taiwan Diabetes Registry (TDR) is a nationwide, multicenter study to assess real-world clinical practices and outcomes for patients with diabetes in Taiwan. The data obtained from the TDR include collection of anthropometric parameters, laboratory examinations, foot assessment and medical records allowing detailed evaluation of diabetes-associated macrovascular and microvascular complications. Participants also completed some questionnaires containing information of sleep duration, sitting time per day and dietary habit with meal frequency. Then, we defined the unhealthy lifestyle behaviors based on previous reports related to three factors: sleep [10], sitting [11] and diet [12, 13]. The severity of unhealthy lifestyle was further stratified by the numbers of disturbed factors and associated with diabetes related vascular comorbidities. Our main purpose is to characterize the risky lifestyle behaviors which potentially pose a threat of vascular complications to subjects with diabetes. Ultimately, we hope that these insights will emphasize the importance of behavioral metabolic control that can be utilized as therapeutic targets to minimize the risks of vascular complications in populations with diabetes.

## Material and methods

### Study design and ethics statement

The Taiwan Diabetes Registry (TDR) is a national, observational study launched by the Diabetes Association of Republic of China (R.O.C., Taiwan) in October 2015 and designed for prospective follow-up with a total of 14 medical centers, 44 regional and local hospitals and 37 general practice clinics participated in the program. Three groups of patients were recruited in the TDR including those with type 1 diabetes mellitus, patients with newly diagnosed (within 6 months) T2DM and patients with long-duration of T2DM who had previously jointed the surveys conducted by the Taiwan Association diabetes educators [17, 18]. Therefore, the patients with long-duration of T2DM have detailed records of previous medical history with regular follow-up (more than 2 outpatient visits per year). In the registration of database, informed consent was obtained, some questionnaires were completed, clinical information and various diabetes-related medical records were collected using an electronic portal (e-portal) web-based platform. This study was approved by the Joint Institute Review Board in Taiwan (protocol number: 14-S-012), and the Institutional Review Board of the Tri-Service General Hospital (TSGHIRB No. 1-104-05-157).

Currently, only cross-sectional data of first registration were released and were utilized in this study to evaluate the association between unhealthy lifestyle behaviors and long-term diabetic vascular complications. To do this, we mainly analyzed dataset from 1188 patients with long-duration of T2DM (13 individuals were excluded due to incomplete or incorrect information). Besides, data of 3285 individuals with newly diagnosed T2DM were also analyzed as the comparison to support the potential long-lasing impacts of dysregulated lifestyle on the diabetes associated vascular comorbidities.

### Data collection

Information included general characteristics of diabetes, personal and family history, past disease history, lifestyle patterns (smoking, drinking, betel nut chewing, diet habit, hours of sleep and daily activity), physical examination (height, weight, blood pressure, waist and hip circumference), laboratory data (blood glucose, HbA1c, lipids, renal and liver function, urine analysis), foot assessment, cardiovascular and microvascular complications of diabetes, diabetes education and self-management status, hospitalization history, and medications used were all documented in the web-based platform of TDR by the diabetes educators and health care providers. Additionally, all the participants completed some questionnaires such as the EuroQol 5-dimension (EQ-5D), the Patient Health Questionnaire-9 (PHQ-9), and the International Physical Activity Questionnaire (IPAQ). The EQ-5D is a 5-dimension questionnaire (mobility, self-care, usual activities, pain/discomfort, anxiety/depression) developed by the EuroQol group in the 1980s to assess health-related quality of life (QOL) [19]. The other questionnaire, PHQ-9, contains 9 items and is one of the easy tools used to screen for the presence and severity of depression and to monitor response to treatment [20]. Then, the IPAQ is a comprehensive instrument that can be used internationally to measure health-related physical activity (PA) in populations [21]. The IPAQ can provide practitioners with an estimate of physical activity and sedentary behavior.

### Three factors for stratifying unhealthy lifestyle behaviors

According to the clinical information obtained from the web-based platform and various self-administered questionnaires, three factors including sleep duration, sit duration, and meals numbers with night snack ingestion were utilized to assess lifestyle behaviors of study subjects. Based on previous reports [10–13], sleep duration for less than 7 h or more than 9 h per day, sit duration for ≥ 8 h a day, and ingestion for ≥ 4 meals with night snack were defined as dysregulated lifestyle in this study. Patients with 0 factor, 1 factor and ≥ 2 factors of unhealthy lifestyle behaviors were stratified to assess their prevalence and associations with diabetes related macro- and micro-vascular diseases. The role of each factor on different vascular comorbidity was further assessed as well.

### Definition of vascular complications of diabetes

Macrovascular complications are composed of cardiovascular disease, cerebrovascular disease, and peripheral arterial occlusive disease (PAOD); whereas microvascular complications comprise neuropathy, nephropathy, and retinopathy [1, 2]. In this study, patients with any one of the listed conditions including coronary heart disease, myocardial ischemia and/or infarction, angina, congestive heart failure, arrhythmia, and a history of percutaneous transluminal coronary angioplasty (PTCA) or coronary artery bypass graft surgery (CABG) were referred to have cardiovascular disease. The cerebrovascular disease is defined as a group of diseases including transient ischemic attack (TIA), ischemic stroke, and hemorrhagic stroke. PAOD is defined as a composite of following status, such as having symptom of intermittent claudication, abnormal foot assessment with reduced or absent pulse over dorsalis pedis artery and/or posterior tibial artery, and a history of percutaneous transluminal angioplasty (PTA), peripheral artery bypass surgery, or amputation. Moreover, diabetic polyneuropathy comprises patients who had neurologic symptoms or aberrant neurologic physical examinations such as decrease/loss of vibratory or pinprick sensation tested by hemi-quantified tuning fork and single-stranded nylon, respectively, on either foot. Patients with diabetic retinopathy are defined as those who had one of the following conditions including macular degeneration, non-proliferative diabetic retinopathy (NPDR), proliferative diabetic retinopathy (PDR), blindness, or receiving laser therapy of retina in the past. Estimated glomerular filtration rate (eGFR), expressed in ml/min/1.73 m^2^, was calculated using the equation from Modification of Diet in Renal Disease (MDRD) [22]. Finally, diabetic kidney disease (DKD) in this study was defined as eGFR < 60 ml/min/1.73 m^2^ or albuminuria defined as a spot urine albumin to creatinine ratio (UACR) ≥ 30 mg/g.

### Statistical analyses

Statistical analysis was carried out using SPSS Statistics for Windows version 22.0 (IBM SPSS Inc., Chicago, IL, USA). For descriptive analysis, continuous variables were shown as mean and standard deviation (S.D.), and categorical variables were expressed as numbers (n) and percentages (%). The differences of continuous variables among three groups were analyzed using the Kruskal–Wallis test due to non-normal distribution (checked by one-sample Kolmogorov–Smirnov test), and the chi-square test was used to compare the categorical variables. The univariate and multivariate logistic regression models were further applied to analyze the impact of dysregulated lifestyle behaviors to specific vascular complications of T2DM. In the multivariate logistic regression, covariables related to demographic characteristics and socioeconomic status were chosen, particularly those that reach statistical significance in univariate analysis. The crude OR was shown in the univariate logistic regression test, whereas the adjusted OR was referred to the results in the multivariate logistic regression model. Statistical significance was defined as a *p*-value of <0.05.

## Results

A total of 1188 T2DM patients (625 males and 563 females) with mean age of 65.9 years and average DM duration of 14.0 years were enrolled. Table 1 presents the demographic characteristics of the study populations stratified via scoring the number of factors (0 factor, 1 factor, and ≥ 2 factors) that stand for unhealthy lifestyle behaviors. Factors indicating dysregulated lifestyle were referred to sleep duration (sleep < 7 h or > 9 h a day), sit duration (sit for ≥ 8 h per day), and frequency of meals (≥ 4 meals per day with night snack ingestion). Overall, there were 41.1% (n = 488) patients with 0 factor, 45.1% (n = 536) patients with 1 factor, and 13.8% (n = 164) patients with ≥ 2 factors analyzed in this study. Generally, females had more unfavorable dysregulated lifestyle than males (*p* = 0.013). The levels of waist circumference, fasting glucose, glycated hemoglobin, triglyceride and creatinine were significantly increased as having increased number of dysregulated lifestyle behaviors (*p*-value of 0.024, 0.045, 0.019, 0.011, and 0.002, respectively). Additionally, highly educated persons with junior college degree or above were less prone to lifestyle dysregulation (*p* = 0.023). In contrast to the married subjects, patients who were single, divorced or widowed tended to behave in unhealthy life pattern (*p* = 0.021). Notably, it also revealed that patients who took metformin or sulfonylurea (SU) were less likely to have lifestyle dysregulation (*p*-value of 0.016, and 0.006, respectively), while patients who used glucagon-like peptide 1 receptor agonist (GLP1RA) and insulin injection as therapeutic agents of T2DM had more dysregulated lifestyle behaviors (*p*-value of 0.011 and 0.001, respectively). However, there was no difference between the three subgroups in receiving other oral hypoglycemic agents, including alpha-glucosidase inhibitor (AGI), thiazolidinedione (TZD), sodium-glucose co-transporter 2 inhibitor (SGLT2i), and dipeptidyl peptidase 4 inhibitor (DPP4i). Regarding residence in urban (capital city) or rural areas, the three subgroups also did not reach statistical differences.

**Table 1:** Basic characteristics of long-duration T2DM patients divided by number of factors that stand for unhealthy lifestyle

	All	0 factor	1 factor	≥ 2 factors	*p*-value
	(n = 1188)	(n = 488)	(n = 536)	(n = 164)	
Age (years)	65.9 (11.4)	65.8 (10.8)	65.5 (11.5)	67.5 (12.9)	0.191
Male % (n)	52.6% (625)	57.4% (280)	50.4% (270)	45.7% (75)	0.013*
BMI (kg/m^2^)	26.2 (4.2)	26.0 (4.0)	26.3 (4.1)	26.8 (4.7)	0.103
Waist (cm)	90.8 (10.6)	90.1 (9.9)	90.7 (10.8)	93.0 (12.0)	0.024*
Waist-to-hip ratio	0.93 (0.07)	0.93 (0.07)	0.93 (0.07)	0.94 (0.08)	0.182
Systolic BP (mmHg)	132.8 (16.3)	132 (16)	133 (16)	136 (20)	0.105
Diastolic BP (mmHg)	74.4 (10.6)	74 (10)	74 (10)	76 (13)	0.058
Fasting glucose (mg/dL)	141.7 (46.2)	140 (46)	141 (44)	151 (55)	0.045*
HbA1c (%)	7.6 (1.3)	7.5 (1.3)	7.6 (1.2)	7.8 (1.5)	0.019*
LDL cholesterol (mg/dL)	89.9 (26.4)	89 (25)	90 (27)	92 (29)	0.704
Triglyceride (mg/dL)	133.2 (104.2)	127 (89)	134 (115)	149 (109)	0.011*
Creatinine (mg/dL)	1.1 (0.9)	1.1 (0.9)	1.1 (0.9)	1.3 (1.1)	0.002**
ALT (U/L)	28.7 (21.1)	28 (21)	29 (20)	29 (24)	0.718
Years of diabetes	14.0 (8.5)	13.9 (8.4)	13.7 (8.3)	15.3 (9.3)	0.252
Smoking % (n)	28.0% (333)	29.1% (142)	26.5% (142)	29.9% (49)	0.554
Alcohol drinking % (n)	19.2% (228)	22.1% (108)	17.2% (92)	17.1% (28)	0.100
Low education % (n)	5.2% (62)	4.7% (23)	5.2% (28)	6.7% (11)	0.611
High education % (n)	16.9% (201)	20.3% (99)	15.3% (82)	12.2% (20)	0.023*
Capital residence % (n)	8.8% (104)	8.8% (43)	8.6% (46)	9.1% (15)	0.974
Married % (n)	83.7% (994)	84.6% (413)	85.1% (456)	76.2% (125)	0.021*
Statin % (n)	48.8% (551)	45.1% (220)	47.8% (256)	45.7% (75)	0.696
Metformin % (n)	74.9% (846)	71.7% (350)	73.1% (392)	63.4% (104)	0.016*
AGI % (n)	12.3% (139)	11.3% (55)	12.5% (67)	10.4% (17)	0.692
SU % (n)	51.9% (586)	48% (234)	53.4% (286)	40.2% (66)	0.006**
TZD % (n)	7.7% (87)	7% (34)	8.2% (44)	5.5% (9)	0.455
SGLT2i % (n)	3.4% (38)	2% (10)	3.7% (20)	4.9% (8)	0.141
DPP4i % (n)	38.4% (434)	38.1% (186)	35.8% (192)	34.1% (56)	0.486
GLP1RA % (n)	1.3% (15)	0.6% (3)	1.1% (6)	3.7% (6)	0.011*
Insulin % (n)	28.5% (338)	26.8% (131)	26.3% (141)	40.2% (66)	0.001**

Number of factors standing for unhealthy lifestyle: scoring by sleep duration, sit duration and frequency of meals and night snack. Continuous variables were analyzed using the Kruskal–Wallis test and are presented as mean values and (standard deviation); Categorical variables were analyzed using the Chi-square test and are presented as percentages (number)

BMI, body mass index; BP, blood pressure; HbA1c, glycated hemoglobin; LDL, low density lipoprotein; ALT, alanine aminotransferase; AGI, alpha glucosidase inhibitor; SU, sulfonylurea; TZD, thiazolidinedione; SGLT2i, sodium-glucose co-transporter 2 inhibitor; DPP4i, dipeptidyl peptidase 4 inhibitor; GLP1RA, glucagon-like peptide 1 receptor agonist

*p < 0.05; **p < 0.01; the bold values refer to variables with *p* < 0.05

Since patients with more dysregulated lifestyle behaviors presented central fat accumulation, increased levels of triglyceride, creatinine and less adequate glycemic control as shown in Table 1, we further assessed whether increased number of factors that disturbed normal lifestyle linked to elevated prevalence of macro- and micro-vascular comorbidities in patients with long-duration of T2DM as illustrated in Table 2. Notably, the prevalence of cardiovascular disease are doubling in patients with ≥ 2 factors as comparing to the prevalence rate in those with 0 factor or 1 factor (*p*-value of 0.001). Similarly, the risk of peripheral arterial occlusion disease (PAOD) and nephropathy were significantly elevated among individuals with higher number of factors (*p*-valuate of 0.014 and 0.037, respectively). While there is no statistically significant difference on the prevalence of cerebrovascular disease, polyneuropathy, and retinopathy among these three subgroups. Then, we further performed univariate and multivariate logistic regression to evaluate the associations between the number of dysregulated lifestyle factors and cardiovascular disease, PAOD, and nephropathy. Univariable logistic regression analysis disclosed that patients with ≥ 2 factors of lifestyle dysregulation presented significantly increased associations with the development of cardiovascular disease, PAOD, and nephropathy with a crude odds ratio (OR) of 2.51 (95% confidence interval (CI) 1.47–4.28, *p* = 0.001) for cardiovascular disease, crude OR of 2.94 (95% CI 1.39–6.24, *p* = 0.005) for PAOD, and crude OR of 1.46 (95% CI 1.02–2.09, *p* = 0.040) for nephropathy (Table 3). Particularly, after adjustment of multiple variables including age, gender, BMI, DM duration, married or not, history of smoking, history of alcohol drinking, education status, income status and living in capital city or not, patients with more than 2 unfavorable lifestyle behaviors still maintained significantly higher probability of cardiovascular disease and PAOD, with an adjusted OR of 2.09 (95% CI 1.18–3.69, *p* = 0.012) and 2.68 (95% CI 1.21–5.90, *p* = 0.015), respectively (Table 3). For further demonstrating the existence of unhealthy lifestyle at the early stage of T2DM and its potential long-lasting impacts, the same analysis was conducted in 3285 individuals with newly diagnosed T2DM from the TDR data as shown in Additional file 1: Tables S1 and S2. Intriguingly, we found the prevalence rates of 0 factor, 1 factor, and ≥ 2 factors of unhealthy lifestyle are very similar among patients with long-duration and newly diagnosed of T2DM with respective 41.1% and 39.6% for 0 factor, 45.1% and 46.1% for 1 factor, and 13.8% and 14.3% for ≥ 2 factors (Additional file 1: Table S1). Whereas the associations between the number of dysregulated lifestyle factors and cardiovascular disease, PAOD, and nephropathy could not be observed in these patients with newly diagnosed T2DM (Additional file 1: Table S2). These results indicated the unfavorable 24-h physical behaviors already existed at the initial diagnosis of T2DM and likely have important long-lasting influence on the subsequent development of diabetes associated vascular complications.

**Table 2:** Prevalence of diabetes associated comorbidities in long-duration T2DM patients divided by number of factors that stand for unhealthy lifestyle

	0 factor	1 factor	≥ 2 factors	*p*-value
	(n = 488)	(n = 536)	(n = 164)	
*DM associated comorbidities*				
Cardiovascular disease % (n)	7.6% (37)	7.8% (42)	16.5% (27)	0.001**
Cerebrovascular disease % (n)	2.0% (10)	3.0% (16)	4.3% (7)	0.307
PAOD % (n)	3.1% (15)	4.7% (25)	8.5% (14)	0.014*
Polyneuropathy % (n)	12.3% (60)	15.1% (81)	15.2% (25)	0.379
Retinopathy % (n)	19.7% (96)	24.6% (132)	19.5% (32)	0.117
Nephropathy % (n)	36.1% (176)	34.1% (183)	45.1% (74)	0.037*

Number of factors standing for unhealthy lifestyle: scoring by sleep duration, sit duration and frequency of meals and night snack. Categorical variables were analyzed using the Chi-square test and are presented as percentages (number)

PAOD, Peripheral arterial occlusion disease

*p < 0.05; **p < 0.01; the bold values refer to variables with *p* < 0.05

**Table 3:** Odds ratios for cardiovascular disease, PAOD and nephropathy divided by number of factors in patients with long-duration T2DM

	1 factor	≥ 2 factors
	OR (95% CI), *p*-value	OR (95% CI), *p*-value
*Cardiovascular disease*		
Crude OR	1.03 (0.65–1.63), 0.903	2.51 (1.47–4.28), 0.001**
§Adjusted OR	1.05 (0.65–1.70), 0.831	2.09 (1.18–3.69), 0.012*
*PAOD*		
Crude OR	1.54 (0.80–2.96), 0.193	2.94 (1.39–6.24), 0.005**
§Adjusted OR	1.49 (0.76–2.91), 0.248	2.68 (1.21–5.90), 0.015*
*Nephropathy*		
Crude OR	0.92 (0.71–1.19), 0.519	1.46 (1.02–2.09), 0.040*
§Adjusted OR	0.89 (0.69–1.16), 0.404	1.25 (0.86–1.81), 0.251

Number of factors standing for unhealthy lifestyle: scoring by sleep duration, sit duration and frequency of meals and night snack. The group of 0 factor was used as the reference. Univariable (crude OR) and multivariable (adjusted OR) logistic regression were performed

OR, odds ratio; CI, confidence intervals. §Adjusted for age, sex, BMI, DM duration, marital status, education, income, capital residence, smoking status, drinking status

*p < 0.05; **p < 0.01; the bold values refer to variables with *p* < 0.05

In order to understand the potential roles of specific lifestyle dysregulation on associated vascular complications (cardiovascular disease, PAOD, and nephropathy), we further performed univariate and multivariate logistic regression analysis. The favorable lifestyle behaviors matching to sleep duration, sitting time and frequency of meals were respectively used as the reference (Table 4). Remarkably, the disturbed eating behavior with ≥ 4 meals per day and night snack ingestion were highlighted to have strong and specific associations with the occurrence of cardiovascular disease and nephropathy, which were remained significantly even after adjusting several variables using multivariate logistic regression analysis, with an adjusted OR of 2.60 (95% CI 1.28–5.30, *p* = 0.009) for cardiovascular disease and 2.54 (95% CI 1.52–4.26, *p* < 0.001) for nephropathy. Besides, the presence of sedentary lifestyle with sit duration ≥ 8 h a day was solely and significantly associated with PAOD even after multivariable adjustment, with an adjusted OR of 4.32 (95% CI 2.38–7.84, *p* < 0.001). However, T2DM patients with reduced or prolonged sleep duration have no significant associations with the development of cardiovascular disease, PAOD or nephropathy as comparing to those with normal 7 to 9 h sleep duration (Table 4).

**Table 4:** Odds ratios for cardiovascular disease, PAOD and nephropathy divided by individual factor in patients with long-duration T2DM

	Sleep duration	Sit duration	Meal numbers and nigh snack
	(< 7 or > 9 h, n = 557)	(≥ 8 h, n = 245)	(≥ 4 with night snack, n = 68)
	OR (95% CI), *p*-value	OR (95% CI), *p*-value	OR (95% CI), *p*-value
*Cardiovascular disease*			
Crude OR	1.31 (0.88–1.96), 0.185	1.53 (0.97–2.40), 0.067	2.49 (1.28–4.84), 0.007**
§Adjusted OR	1.31 (0.86–1.99), 0.207	1.23 (0.76–2.01), 0.403	2.60 (1.28–5.30), 0.009**
*PAOD*			
Crude OR	0.77 (0.44–1.34), 0.356	4.20 (2.42–7.31), < 0.001**	1.74 (0.67–4.51), 0.258
§Adjusted OR	0.71 (0.40–1.26), 0.242	4.32 (2.38–7.84), < 0.001**	1.69 (0.63–4.53), 0.296
*Nephropathy*			
Crude OR	0.86 (0.68–1.10), 0.227	1.29 (0.97–1.72), 0.082	2.33 (1.42–3.81), 0.001**
§Adjusted OR	0.81 (0.63–1.04), 0.091	1.13 (0.84–1.53), 0.414	2.54 (1.52–4.26), < 0.001**

Sleep duration between 7 and 9 h, sit duration < 8 h, and other frequency of meals (≦3 or ≥ 4 without nigh snack) were used as the reference, respectively. Univariable (crude OR) and multivariable (adjusted OR) logistic regression were performed

OR, odds ratio; CI, confidence intervals. §Adjusted for age, sex, BMI, DM duration, marital status, education, income, capital residence, smoking status, drinking status

*p < 0.05; **p < 0.01; the bold values refer to variables with *p* < 0.05

## Discussion

In this cross-sectional analysis of a nationwide, multicenter diabetes registry cohort in Taiwan, we revealed both long-duration (n = 1188) and newly diagnosed (n = 3285) T2DM patients presented high prevalence of unhealthy lifestyle with around 60% of those possessed at least one unfavorable 24-h lifestyle behavior as counting the daily sleep duration, sitting time and meal frequency. Therefore, the status of dysregulated lifestyle should be evaluated and corrected as soon as T2DM was diagnosed. We also found some subgroups of long-duration T2DM patients are less prone for severe lifestyle dysregulation including males, highly educated persons and those who are married, suggesting these unfavorable lifestyle behaviors are related to the socioeconomic status and might be adjustable through education. We also found having more factors disturbing normal lifestyle behaviors was associated with several detrimental cardiometabolic parameters, such as increased waist circumference, higher levels of triglyceride, creatinine and poor glycemic control. More importantly, the associations between unhealthy lifestyle and diabetes related vascular comorbidities could only be observed in patients with long-duration of T2DM, indicating these unfavorable lifestyle behaviors are likely to have long-lasting impacts for the development of vascular complications in T2DM patients.

To the best of our knowledge, this was the first study trying to investigate the associations between lifestyle behaviors and vascular complications of diabetes. Our data showed there is significantly increased prevalence and associations of cardiovascular disease, PAOD and nephropathy in long-duration T2DM patients having ≥ 2 risk factors of lifestyle dysregulation. Whereas only cardiovascular disease and PAOD remained statistically significant associations after adjusting covariables. Overall, our results highlighted that unhealthy lifestyle could not only deteriorate the cardiometabolic parameters, but also contribute to the incidences of cardiovascular illnesses in T2DM. Theoretically, it seems to be very reasonable because diabetes and metabolic syndrome are well-established risks for cardiovascular disease. Notably, several types of adverse cardiovascular events including unstable angina [23], acute myocardial infarction [23, 24], sudden cardiac death [24, 25] were shown to present circadian variation with the highest incidence occurs in the awaking daytime. The morning peak in adverse cardiovascular events may relate to the beginning of awaking activity [23], increased sympathetic tone, more aggregable platelets with increased coagulation, more vulnerable atherosclerotic plaque, and higher plasma cortisol and epinephrine levels [23, 26]. Additionally, dysregulated lifestyle behaviors associated circadian disruption also increases inflammatory markers including CRP [27, 28], TNF-alpha [27, 28], IL-6 [27], IL-10 [28], and resistin [27]. Moreover, a recent prospective investigation in patients with ST-segment elevation myocardial infarction further demonstrated that dysregulated lifestyle such as shift work was associated with augmented reperfusion injury and increased risks of major adverse cardiac events during a median follow-up of 5.0 years [14]. Collectively, the presence of lifestyle dysregulation conveys pleiotropic effects on triggering cardiovascular diseases, thereby providing a pathophysiologic link between unhealthy lifestyle and vascular complications in patients with long-term T2DM as observed in this study.

Moreover, our study also found that specific lifestyle dysregulation was associated with specific vascular complication in patients with T2DM. Those with unfavorable eating habits had higher probabilities for developing cardiovascular disease and nephropathy, whereas the sedentary lifestyle was significantly associated with PAOD. In this study, we defined the misaligned eating behavior as meal frequency with ≥ 4 meals per day along with night snack intake, which likely combines the mixed effect of increased eating occasions, delayed eating time with night supper ingestion, and possible irregular eating schedule. Although light is the primary zeitgeber of the central clock [29], peripheral clocks derived from various peripheral organs are influenced by food supply [30]. A recent study disclosed the timing of nutrient delivery could influence the cardiometabolic health via alterations in peripheral clocks, most notably in the liver [31]. In other words, food is one of the external synchronizers of peripheral clocks. For example, changes in the timing and frequency of food intake may lead to an uncoupling of peripheral oscillators from the central pacemaker. As a result, the feeding/fasting behavior entrains clock genes and regulates various aspects of metabolism. Indeed, many studies have proved that disturbed eating time and frequency can trigger the development of obesity [30, 32], type 2 diabetes [32], and cardiovascular disease [33].

Although how unhealthy eating habit steers the increased cardiometabolic diseases remains obscure, various hormones and enzymes are likely engaged. Unusual feeding pattern may modify the circadian rhythm via hormones involved in metabolism, including insulin, glucagon, cortisol, adiponectin, and leptin [30]. Liver genes also encode enzymes involved in food processing and expressed in a rhythmic pattern [34]. Adipose tissues also participated in regulating circadian clock network. Some glucocorticoid metabolism-related genes and the transcription factor peroxisome proliferator activated receptor γ (PPARγ), are part of the intrinsic clock controlled genes [35]. Besides, a large number of intestinal enzymes and hormones are also exhibited in a circadian manner and are synchronized by food [30]. Among these, incretins as diet induced gut peptides must be mentioned. The incretin hormone glucagon-like peptide-1 (GLP-1) was physiologically secreted from intestine upon eating and functions to augment insulin secretion, prolong gastric emptying and inhibit appetite [36]. A recent animal study further disclosed the circadian release of GLP-1 was regulated by the core clock gene, *Bmal1* [37]. Importantly, GLP-1 receptor agonist as an injectable anti-diabetic medication has been demonstrated to conduct effective body weight loss along with protective effects for major adverse cardiovascular events and diabetic nephropathy [38]. Therefore, our findings further emphasize the importance of incretin-based therapy in T2DM. Taken together, changes of feeding time/frequency or ingestion of high-energy meals may alter the rhythmic clocks on multiple organs and have decisive influences on cardiometabolic consequences in T2DM.

Last but not least, our data also showed that sedentary lifestyle with sitting duration for more than 8 h a day was significantly associated with PAOD. The causal relationship between long sitting time and PAOD is still uncertain. Currently, only few studies investigated the status of sedentary lifestyle and its impact on people with PAOD. Based on a 5-year follow-up study, the incidence of mobility loss and unable to continuously walk for 6 min was significantly increased in people with PAOD compared to persons who have a normal value of ankle-brachial index [39]. Similarly, greater sitting hours per day and slower outdoor walking speed were found to be associated with faster annual decline in distance of 6-min walk and calf muscle, respectively, in participants with PAOD [40]. Therefore, it is likely that the long sitting behavior might be a consequence of muscular functional impairment happened in patients with PAOD. Moreover, early identification and modification of the sedentary behavior might be important for ameliorating the functional decline in patients with PAOD.

In this study, there are still several limitations. First, this is a cross-sectional analysis, and the causal relationship cannot be ascertained. Second, other behaviors (e.g., exercise strength/frequency/duration and the work pattern with different light/dark cycle) can also interact with the intrinsic clock network and contribute to the cardiovascular diseases but are not included in our investigation. Third, in evaluating the sleep status, we merely counted the average sleep duration without assessing the sleep quality or social jet lag. Finally, we did not obtain the information about food contents from participants during our analysis. We only focused on the meal frequency with or without night snack consumption rather than dietary profiles such as total calories and percentage of macronutrients intake. As a result, some attentions should be given while interpreting our results. The major strength of current study is using a nationwide, multicenter diabetes registry cohort under a web-based platform. Second, we concurrently assess the status of lifestyle dysregulation and its associations with macro- and micro-vascular complications in two groups of patients (long-duration and newly diagnosed T2DM). This approach emphasized the potential long-term impact of unhealthy lifestyle behaviors on vascular comorbidities in T2DM.

In conclusion, the present study showed that unhealthy lifestyle is associated with increased prevalence of diabetic macrovascular and microvascular complications in Taiwanese population. Specifically, patients with disturbed eating habits had higher probabilities of developing cardiovascular disease and nephropathy, whereas the sedentary lifestyle was significantly associated with PAOD. Further research is still needed to understand the mechanisms, the directions of causality and the practical roles to reduce the diabetes associated vascular complications via correcting the dysregulated lifestyle behaviors.

## Supplementary Information

**Additional file 1. Table S1. **Prevalence of factors that stand for unhealthy lifestyle among long-duration and newly diagnosed T2DM patients. **Table S2. **Odds ratios for cardiovascular disease, PAOD, and nephropathy divided by number of factors in patients with newly diagnosed T2DM.

## Data Availability

The datasets used and analyzed during the current study are available from the corresponding author on reasonable request.
