# Supplementary material for: Unhealthy lifestyle associated with increased risk of macro- and micro-vascular comorbidities in patients with long-duration type 2 diabetes: results from the Taiwan Diabetes Registry
Source: Diabetol Metab Syndr. 2023 Mar 8;15:38. doi: 10.1186/s13098-023-01018-9 (PMC9996995; doi:10.1186/s13098-023-01018-9)
Supplement: Supplementary file 1 — Additional file 1. Table S1. Prevalence of factors that stand for unhealthy lifestyle among long-duration and newly diagnosed T2DM patients. Table S2. Odds ratios for cardiovascular disease, PAOD, and nephropathy divided by number of factors in patients with newly diagnosed T2DM. [file 13098_2023_1018_MOESM1_ESM.docx]

**Supplementary Table 1.**

Prevalence of factors that stand for unhealthy lifestyle among long-duration and newly diagnosed T2DM patients

|  |  | Long T2DM | New T2DM | *p* value |
| --- | --- | --- | --- | --- |
|  |  | (n = 1188) | (n = 3285) |  |
| 0 factor  1 factor |  | 41.1% (488)  45.1 % (536) | 39.6 % (1301)  46.1 % (1514) | 0.374  0.565 |
| ≧2 factors |  | 13.8 % (164) | 14.3 % (470) | 0.670 |

Number of factors standing for unhealthy lifestyle: scoring by sleep duration, sit duration and frequency of meals & night snack. Categorical variables were analyzed using the Chi-square test and are presented as percentages (number). **p*<0.05; ***p*<0.01

**Supplementary Table 2.**

Odds ratios for cardiovascular disease, PAOD, and nephropathy divided by number of factors in patients with newly diagnosed T2DM

|  |  | 1 factor  OR (95% CI), *p* value | | ≧2 factors  OR (95% CI), *p* value |  |
| --- | --- | --- | --- | --- | --- |
| **Cardiovascular disease**  Crude OR  §Adjusted OR  **PAOD**  Crude OR  §Adjusted OR |  | | 0.88 (0.59-1.31), 0.523  0.92 (0.62-1.38), 0.697  1.15 (0.71-1.86), 0.568  1.13 (0.70-1.83), 0.621 | 1.11 (0.66-1.88), 0.698  1.09 (0.62-1.90), 0.765  0.83 (0.39-1.76), 0.621  0.81 (0.38-1.73), 0.587 | |
| **Nephropathy** |  |  | |  |  |
| Crude OR |  | 0.88 (0.73-1.07), 0.190 | | 0.97 (0.74-1.27), 0.816 |  |
| §Adjusted OR |  | 0.88 (0.72-1.06), 0.175 | | 0.99 (0.75-1.30), 0.944 |  |

Number of factors standing for unhealthy lifestyle: scoring by sleep duration, sit duration and frequency of meals & night snack. The group of 0 factor was used as the reference. Univariable (crude OR) and multivariable (adjusted OR) logistic regression were performed. Abbreviation: OR, odds ratio; CI, confidence intervals. §Adjusted for age, sex, BMI, marital status, education, income, capital residence, smoking status, drinking status. **p*<0.05; ***p*<0.01
